# Stable naphthalene diimide zwitterions for aqueous organic redox flow batteries

**DOI:** 10.1093/nsr/nwaf286

**Published:** 2025-08-11

**Authors:** Haochen Li, Dawei Feng

**Affiliations:** Department of Materials Science and Engineering, University of Wisconsin-Madison, USA; Department of Materials Science and Engineering, University of Wisconsin-Madison, USA

Aqueous organic redox flow batteries (AORFBs) are essential for safe, large-scale and sustainable grid energy storage. Among the redox-active molecules, naphthalene diimide (NDI) derivatives have emerged as promising anolytes due to their stable two-electron redox properties and tunable solubility [1–3]. However, most of these derivatives still rely on expensive salts, which limits their practical scalability, complicates synthesis and they suffer from irreversible decomposition as the pH increases.

**Figure 1.**
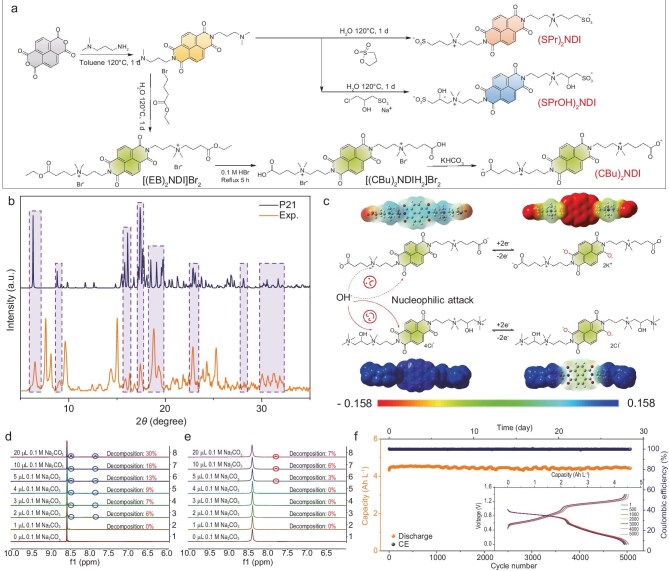
(a) Synthesis route of (SPr)_2_NDI, (SPrOH)_2_NDI and (CBu)_2_NDI. (b) X-ray diffraction (XRD) patterns of (CBu)_2_NDI, experimental data (bottom) and simulation (top). (c) Electrostatic potential of (CBu)_2_NDI and Dex-NDI. The initial proton nuclear magnetic resonance (^1^H NMR) spectra of 0.01 M (d) Dex-NDI and (e) (CBu)_2_NDI at different 0.1 M Na_2_CO_3_ amounts. (f) The 0.1 M (0.2 M e^−^) (CBu)_2_NDI/K_4_Fe(CN)_6_-based AORFB was galvanostatically cycled at 40 mA cm^−2^ (inset: the charge and discharge curves of the 1st, 500th, 1000th, 2000th, 3000th, 4000th, 5000th). Adapted from [4] with permission.

In a recent paper, Prof. Gang He's group [4] synthesized (CBu)₂NDI with a zwitterionic side chain by using an atmospheric pressure method (Fig. 1a). Both experimental and simulation results illustrate that (CBu)₂NDI adopts a distinct parallel-staggered stacking pattern with a face-to-face distance of 3.45 Å and a displacement stacking angle of 42.8° (Fig. 1b). This configuration results from π–π interactions between the naphthalene diimide core and mutual repulsion between the side chains, stabilizing the molecular conformation and achieving high solubility.

Additionally, compared with Dex-NDI, which only has cationic side chains, the introduction of a carboxyl group in (CBu)₂NDI increases the electron density of the naphthalene core, helping to mitigate nucleophilic attack and reduce decomposition (Fig. 1d and e). Proton nuclear magnetic resonance (^1^H NMR) results in D₂O with various amounts of Na₂CO₃ verified that (CBu)₂NDI exhibits significantly lower decomposition under identical conditions.

To further confirm its stability and practical applicability, the team conducted full battery tests by using the synthesized material as an anolyte coupled with K₄Fe(CN)₆ as a catholyte. The results revealed an actual capacity of 4.51 Ah L⁻¹ (theoretical capacity: 5.36 Ah L⁻¹), corresponding to a high-capacity utilization rate of 84.14%. Remarkably, the battery retained 100% capacity after 5070 cycles, with a capacity-fading rate of 0% per cycle.

Finally, the total cost of the electrolytes was calculated. The cost of the batteries based on (CBu)₂NDI/K₄Fe(CN)₆ is as low as $6.58 per Ah in the laboratory—only about 1/5 to 1/20 of the cost of other electrolytes typically used in AORFBs [1–3,5]. The introduction of a zwitterionic design represents a breakthrough at the technological frontier, effectively addressing capacity-fade issues during long-term cycling in flow batteries. This advancement establishes the system's international leadership position, while its ultra-low cost further demonstrates significant commercial viability.

In summary, this work innovatively incorporates zwitterions into naphthalene diimide, comprehensively optimizing its properties and opening up a new pathway for the application of neutral AORFBs in large-scale energy storage.
